# Chronic pathophysiological changes in the normal brain parenchyma caused by radiotherapy accelerate glioma progression

**DOI:** 10.1038/s41598-021-01475-0

**Published:** 2021-11-11

**Authors:** Yuichiro Tsuji, Naosuke Nonoguchi, Daisuke Okuzaki, Yusuke Wada, Daisuke Motooka, Yuki Hirota, Taichiro Toho, Nobuhiko Yoshikawa, Motomasa Furuse, Shinji Kawabata, Shin-Ichi Miyatake, Hiroyuki Nakamura, Ryohei Yamamoto, Shota Nakamura, Toshihiko Kuroiwa, Masahiko Wanibuchi

**Affiliations:** 1 Department of Neurosurgery, Osaka Medical and Pharmaceutical University, 2-7 Daigaku-Machi, Takatsuki, Osaka 569-8686 Japan; 2 Genome Information Research Center, Research Institute for Microbial Diseases, Osaka University, 3-1 Yamadaoka, Suita, Osaka 565-0871 Japan; 3 Department of Advanced Pathobiology, Graduate School of Life and Environmental Sciences, Osaka Prefecture University, 1-58 Rinku Ourai-Kita, Izumisano, Osaka 598-8531 Japan; 4 Department of Radiology, Osaka Medical and Pharmaceutical University, Osaka, Japan; 5 Laboratory for Chemistry and Life Science, Institute of Innovative Research, Tokyo Institute of Technology, 4259 Nagatsuta-cho, Midori-ku, Yokohama, Kanagawa 226-8503 Japan; 6 Division for Advanced Medical Development, Cancer Center, Osaka Medical and Pharmaceutical University, 2-7 Daigaku-Machi, Takatsuki, Osaka 569-8686 Japan; 7 Department of Neurosurgery, Tesseikai Neurosurgical Hospital, 28-1, Nakanohommachi, Shijyonawate, Osaka 575-8511 Japan

**Keywords:** Cancer, CNS cancer, Cancer microenvironment, Radiotherapy

## Abstract

Radiation therapy is one of standard treatment for malignant glioma after surgery. The microenvironment after irradiation is considered not to be suitable for the survival of tumor cells (tumor bed effect). This study investigated whether the effect of changes in the microenvironment of parenchymal brain tissue caused by radiotherapy affect the recurrence and progression of glioma. 65-Gy irradiation had been applied to the right hemisphere of Fisher rats. After 3 months from irradiation, we extracted RNA and protein from the irradiated rat brain. To study effects of proteins extracted from the brains, we performed WST-8 assay and tube formation assay in vitro. Cytokine production were investigated for qPCR. Additionally, we transplanted glioma cell into the irradiated and sham animals and the median survival time of F98 transplanted rats was also examined in vivo. Immunohistochemical analyses and invasiveness of implanted tumor were evaluated. X-ray irradiation promoted the secretion of cytokines such as CXCL12, VEGF-A, TGF-β1 and TNFα from the irradiated brain. Proteins extracted from the irradiated brain promoted the proliferation and angiogenic activity of F98 glioma cells. Glioma cells implanted in the irradiated brains showed significantly high proliferation, angiogenesis and invasive ability, and the post-irradiation F98 tumor-implanted rats showed a shorter median survival time compared to the Sham-irradiation group. The current study suggests that the microenvironment around the brain tissue in the chronic phase after exposure to X-ray radiation becomes suitable for glioma cell growth and invasion.

## Introduction

Initially, the standard treatment of malignant glioma involved surgical removal of as many tumors as possible followed by radiotherapy. Although progression-free survival of glioblastoma is approximately ten months^1^, it has been demonstrated that X-ray treatment reduces the recurrence of tumor and increases the life expectancy^2,3^. To prevent the growth of tumor cells invading tumor-surrounding brain tissue, radiotherapy is targeted at the area within 3–4 cm of the tumor resection cavity^4^. Nevertheless, the recurrence of more than 95% of glioma has been observed in the area within 2–3 cm of the tumor resection cavity at several months or years after radiotherapy^5,6^.

It is known that the implantation of tumor cells into the radiation-exposed tissue delays the onset and reduces the growth rate of tumors^7^. The effect, which was later named as the tumor bed effect^8^, is considered to be the main cause of reduced neovascularization^9,10^. In general, the radiation-exposed tissue environment, which is characterized by reduced blood perfusion (nasal CPAP)^11^, reduced extracellular pH^11^, and a hypoxic environment^12^, is considered to be the microenvironment that is not suitable for the survival of tumor cells.

Other authors reported that radiation stimulated an infiltration of F98 glioma cells into the brains of tumor-bearing Fischer rats, resulting in a reduction of their MST^13^. This stimulation was associated with pro-inflammatory mediators such as cyclooxygenase-2 (COX-2), interleukin-1β (IL-1β), and matrix metalloproteinase-2 (MMP-2). In a related study, the inhibition of inflammatory cytokines by a COX-2 inhibitor prevented F98 glioma progression in the irradiated rat brain and increased the MST^14^. These reports suggested the possibility that tumor growth could paradoxically be stimulated in the acute phase after radiation therapy, but there has been no report discussing the influence of irradiation in the chronic stage on glioma in rodent models.

The tissue microenvironment, in which the regrowth of tumor cells that survived the initial treatment occurs within several months after exposure to the radiation, is the brain tissue different from that of the primary tumor. In this study, we examined the effect of changes in the microenvironment of parenchymal brain tissue (i.e., the tumor bed) in the chronic phase after exposure to X-Ray radiation on F98 glioma cells.

## Results

### Proteins extracted from the irradiated brain promoted the proliferation of F98 cells

**Figure 1:**
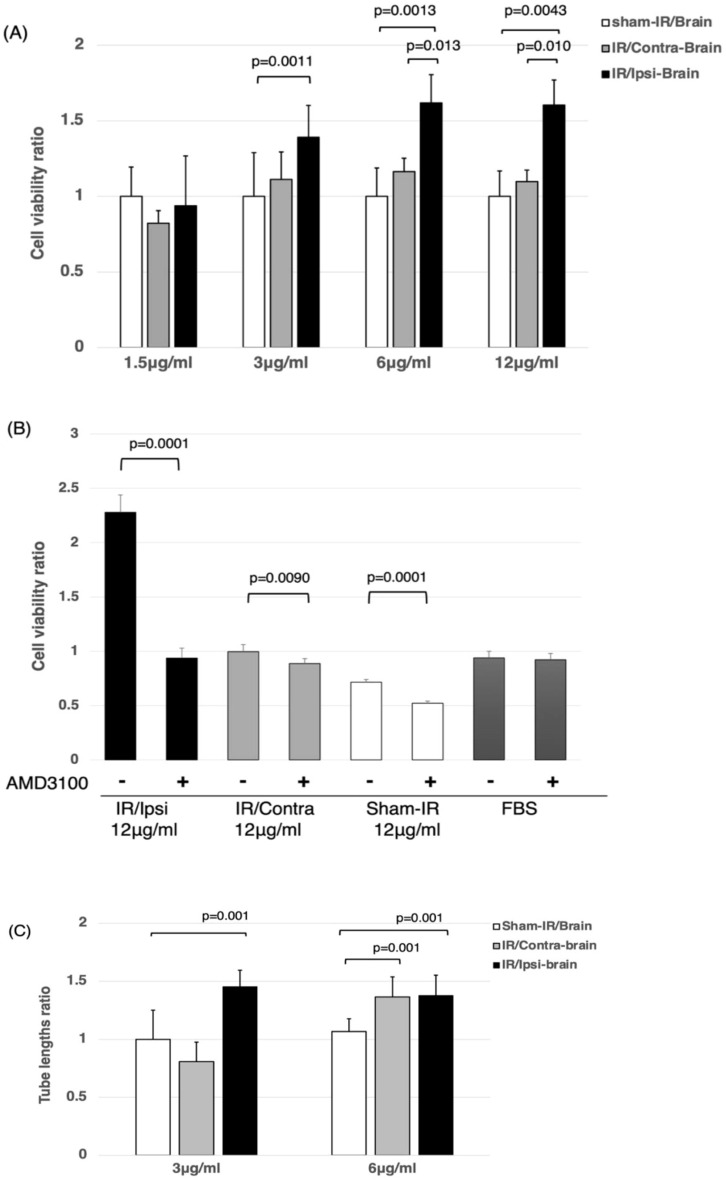
(**A**) The cell-viability ratio under each protein concentration extracted from each group by WST-8 assay. Tissue proteins extracted from the IR/Ipsi-brain group contained significantly higher levels of growth factors compared to the Sham-IR/Brain group under the 3, 6, and 12 μg/ml concentrations of extracted protein. These results were expressed as mean ± SD and comparisons between groups were assessed by Student’s t-test. (**B**) The cell-viability ratio under 12 μg/ml concentrations of extracted protein from each group adding AMD3100(10 μM) by WST-8 assay. The cell-viability ratio adding AMD3100 in IR/Ipsi-brain, IR/Contra-brain, and Sham-IR/Brain groups was significantly inhibited. These results were expressed as mean ± SD and comparisons between groups were assessed by Student’s t-test. (**C**) Measurement of tube lengths by a tube-forming assay under each protein concentration extracted from each group. Tissue proteins extracted from the IR/Ipsi-brain group contained a significantly higher level of angiogenesis factors compared to those of the Sham-IR/Brain group. Under the protein concentration of 6 μg/ml, significantly longer tube formation was observed in the IR/Contra-brain group compared to the Sham-IR/Brain group. These results were expressed as mean ± SD and comparisons between groups were assessed by Student’s t-test.

The proliferation of F98 cells under different concentrations (1.5, 3, 6 and 12 μg/ml) of protein extracted from the brain hemispheres of the IR/Ipsi-brain, IR/Contra-brain, and Sham-IR/Brain groups was measured by WST-8 assay. As shown in Fig. 1A, the cells incubated with medium containing protein extracted from an irradiated hemisphere (here, the IR/Ipsi-brain group) showed a significantly higher cell growth rate compared to the rate of the control group (i.e., Sham-IR/Brain group) under 3, 6 and 12 μg/ml of extracted protein. There was no significance difference in the cell growth rate between the non-irradiated hemisphere (IR/Contra-brain group) and the control group (Sham-IR/Brain group). Additionally, WST-8 assay adding AMD3100 (10 μM)was performed, and the cell proliferation of F98 glioma under 12 μg/ml of protein extracted from the brain hemispheres of the IR/Ipsi-brain, IR/Contra-brain, and Sham-IR/Brain groups was significantly inhibited (Fig. 1B).

### Tube-forming activity was increased by brain irradiation

The angiogenic activity was assessed by measuring the length of forming tubes under different concentrations (3 and 6 μg/ml) of protein extracted from the IR/Ipsi-brain, IR/Contra-brain, and Sham-IR/Brain rats. As shown in Fig. 1C, the IR/Ipsi-brain group showed significantly longer tube formation compared to the Sham-IR/Brain group under protein concentrations of both 3 and 6 μg/ml. In the IR/Contra-tumor group, significantly longer tube formation was observed compared to the Sham-IR/Brain group only under the 6 μg/ml protein concentration.

### Brain irradiation decreased the MST

**Figure 2:**
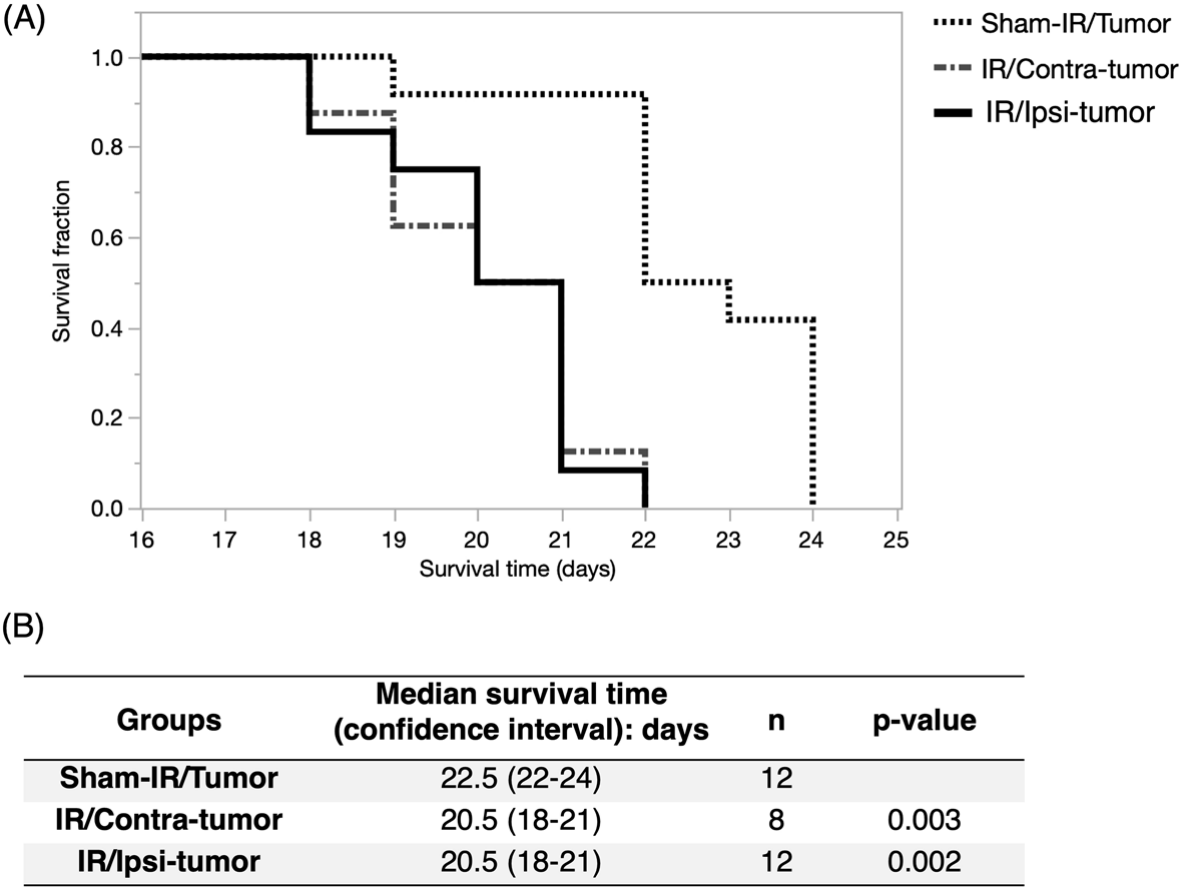
(**A**,**B**) Survival curves of Fischer rats implanted with F98 glioma cells. Significantly shorter median survival times were achieved by the IR/Ipsi-tumor and IR/Contra-tumor rats compared to the Sham-IR/Tumor rats (IR/Ipsi-tumor and IR/Contra-tumor = 20.5 days vs Sham-IR/Tumor group = 22.5 days; p = 0.002 and p = 0.003, respectively). There was no significant difference in survival between the IR/Ipsi-tumor and IR/Contra-tumor groups (p = 0.8).

As shown in Fig. 2A,B, the rats in the IR/Ipsi-tumor and IR/Contra-tumor groups showed significantly shorter MSTs compared to the rats in the Sham-IR/Tumor group. Interestingly, there was no significant difference in survival between the IR/Ipsi-tumor and IR/Contra-tumor groups. The IR/Ipsi-tumor, IR/Contra-tumor, and Sham-IR/Tumor rats all showed good appetites, and there was no difference in the nutritional state among these groups, just before the tumor implantation. We also confirmed that the X-ray irradiated rats without the tumor cell implantation had been living for more than 12 months.

### Changes in tumor biology of rats

**Figure 3:**
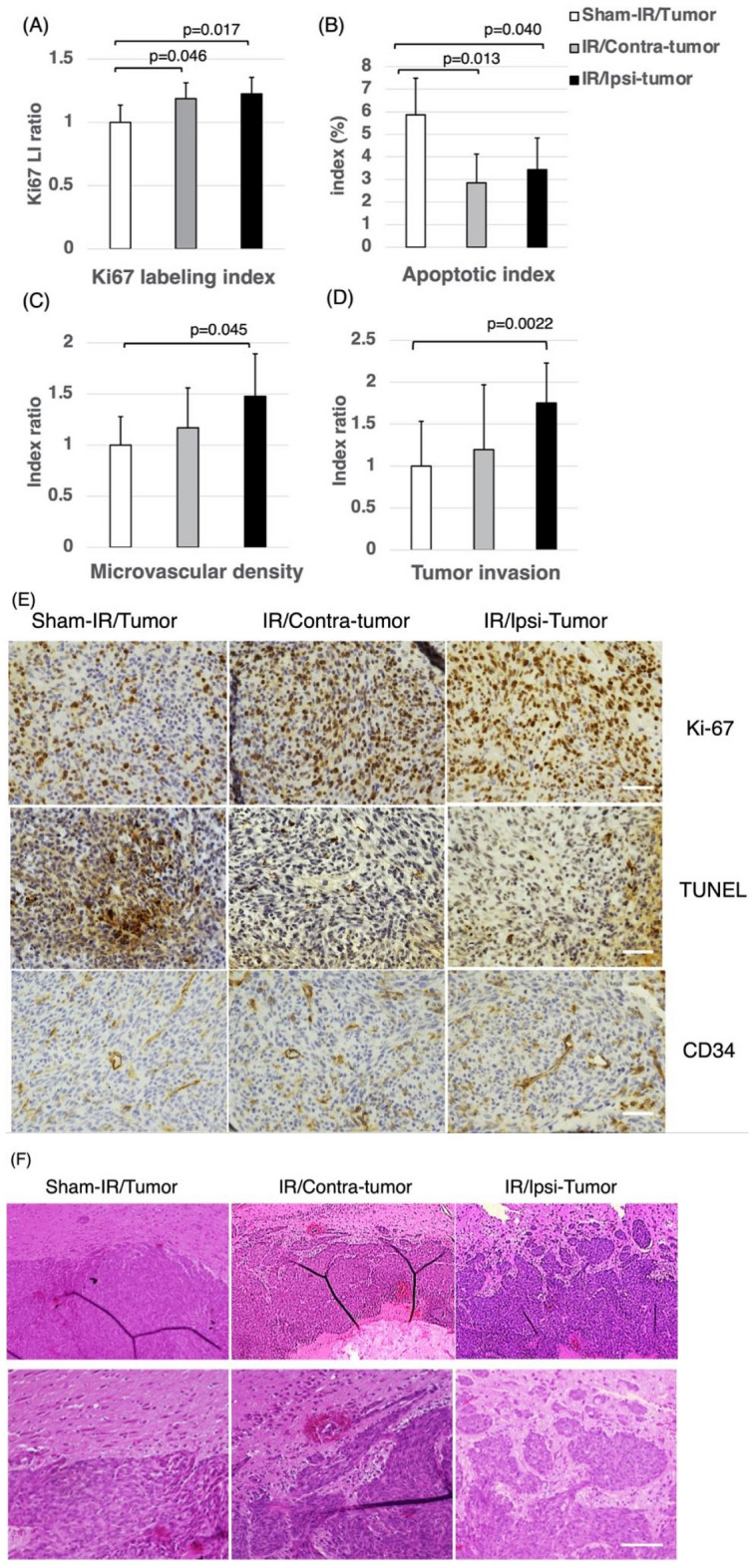
Changes in the biology of the transplanted tumors. (**A**) Ki-67 labeling index. (**B**) Apoptotic index. (**C**) Microvascular density (MVD) index. (**D**) Tumor invasion index. The tumors of the IR/Ipsi-tumor group exhibited significantly higher proliferation, angiogenesis abilities and invasiveness compared to those of the Sham-IR/Tumor group. Tumors of the IR/Ipsi-tumor and IR/Contra-tumor group showed significantly lower rates of apoptosis compared to the Sham-IR/Tumor group. These results were expressed as mean ± SD and comparisons between groups were assessed by Student’s t-test. (**E**) F98 glioma xenografts were stained immunohistochemically for Ki-67 (upper panels), TUNEL (middle panels) and CD34 (lower panels) at a magnification of × 400. Tumors of the IR/Ipsi-tumor and IR/Contra-tumor group showed a significant increase in proliferative index and a significant decrease in apoptotic index compared to Sham/IR-Tumor group. Tumors of the IR/Ipsi-tumor showed a significant increase in microvascular density index compared to Sham/IR-Tumor group. Scale bars = 50 μm. (**F**) Hematoxylin and eosin staining of F98 tumor showed tumor rim for sham-IR/Tumor (left panels), IR/Contra-tumor (middle panels) and IR/Ipsi-tumor (right panels). The upper panel shows the image at a magnification of × 80, and the area surrounded by the square shows the image at a magnification of × 200 (lower panel). Scale bars = 100 μm.

As shown in Fig. 3A,E, the Ki67 labeling index increased significantly in the IR/Ipsi-tumor group compared to the Sham-IR/Tumor group. The Ki67 index indices of the IR/Contra-tumor and Sham-IR/Tumor groups were not significantly different. The apoptotic index of the IR/Contra-tumor group was significantly decreased compared to that of the Sham-IR/Tumor group (Fig. 3B,E). The MVD index of the IR/Ipsi-tumor group was significantly higher than that of the Sham-IR/Tumor group, but there was no significant difference in the MVD index between the IR/Contra-tumor and Sham-IR/Tumor groups (Fig. 3C,E).

### Increased tumor invasion in the irradiated brain

The tumor surface in the IR/Ipsi-tumor group was irregular and the tumors exhibited strong invasiveness. On the other hand, the Sham-IR/Tumor group showed a relatively clear border around the tumor rim. A significant increase in the number of tumor cells invading normal brain tissues was observed in the IR/Ipsi-tumor group compared to the Sham-IR/Tumor group (Fig. 3D,F). There was no significant difference between the IR/Contra-tumor and Sham-IR/Tumor groups.

### Whole transcriptome analysis (RNA-seq) for the irradiated brain

**Figure 4:**
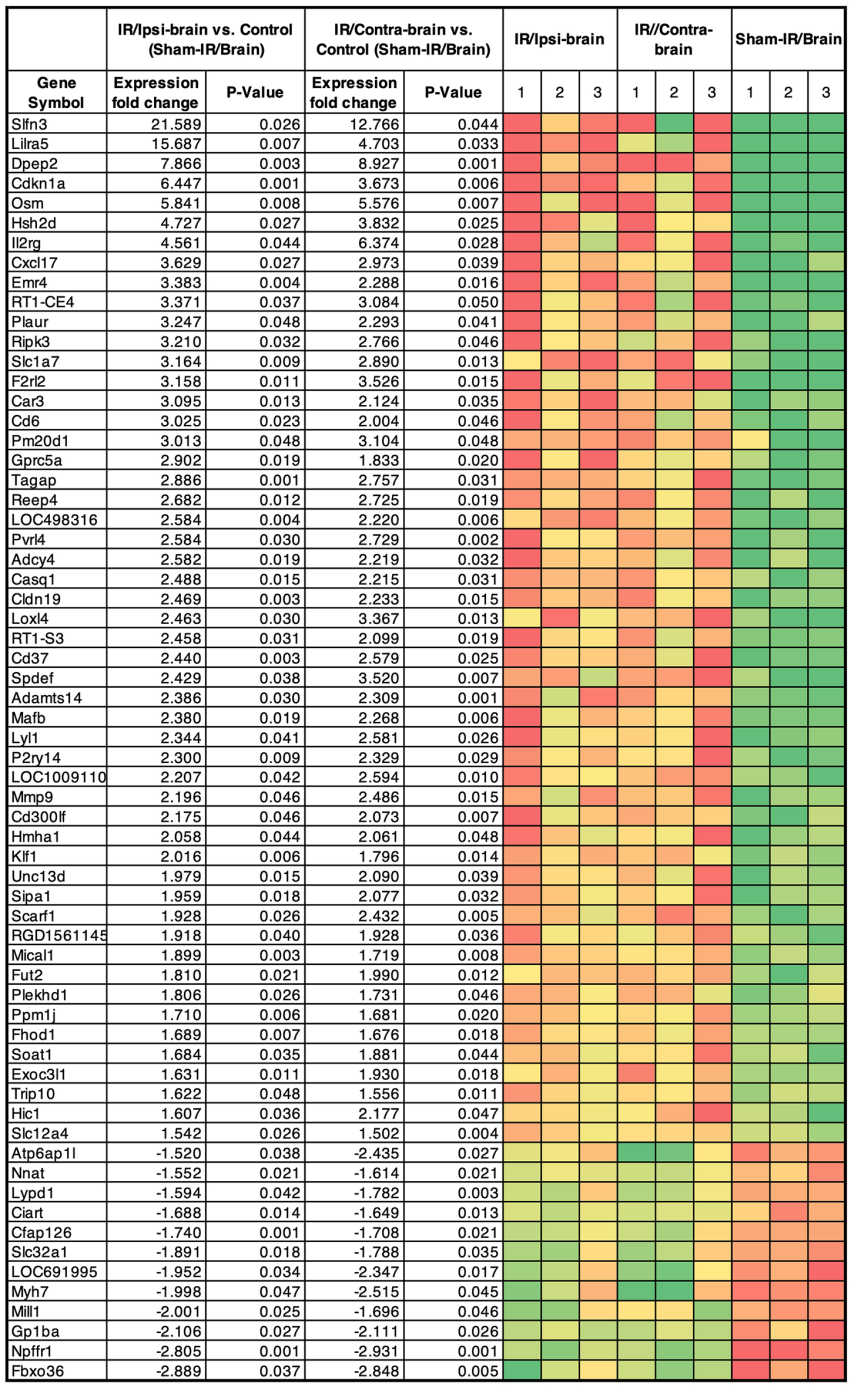
64 genes that showed significant expression changes of more than 1.5-fold or less than − 1.5-fold in both x-ray irradiated and non-irradiated hemisphere, compared to the sham-irradiated brain. Significance level is p < 0.05.

In comparison with the Sham-IR/Brain (control), 64 differentially expressed genes (DEGs) were commonly up- or down-regulated both in the IR/Ipsi-brain and IR/Contra-brain of the irradiated animals, as listed in Fig. 4.

The pathway analysis for the DEGs revealed that 13 pathways were commonly impacted both in IR/Ipsi-brain and IR/Contra-brain groups, and out of these pathways, four pathways involved tissue inflammation, such as cell adhesion molecules (CAMs), chemokine signaling pathway, leukocyte transendothelial migration, and cytokine-cytokine receptor interaction (Table 1).

**Table 1:** The list of the significantly impacted pathways in IR/Ipsi-brain and IR/Contra-brain groups, as compared to the control group (Sham-IR/Brain).

Impacted pathways	IR/Ipsi-brain vs. Sham-IR/Brain		IR/Contra-brain vs. Sham-IR/Brain	
	P-value	Bonferroni correction	P-value	Bonferroni correction
Cell adhesion molecules (CAMs)	1.10E−06	0.00026	0.008	1
Chemokine signaling pathway	9.40E−05	0.024	0.029	1
Leukocyte transendothelial migration	0.00032	0.080	0.0034	0.94
Cytokine-cytokine receptor interaction	0.0012	0.29	0.0071	1

Bonferroni; corrected P-values by the Bonferroni method for multiple comparisons.

### Stimulation of genes and molecules in the brain by irradiation

**Figure 5:**
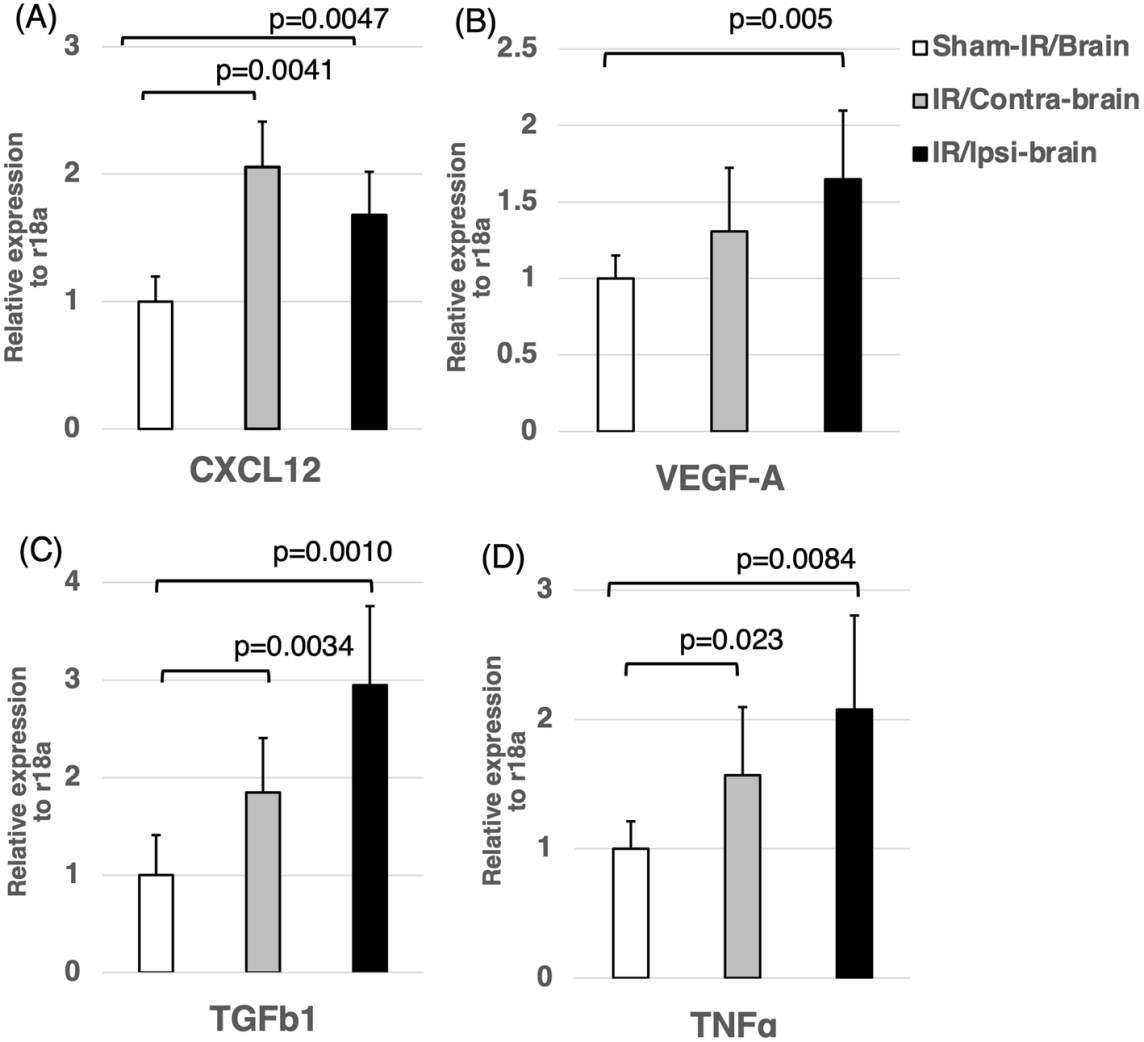
Expression of various cytokines in the IR/Ipsi-brain, IR/Contra-brain, and Sham-IR/Brain groups. The expressions of CXCL12, VEGF-A, TGF-β1 and TNFα in the IR/Ipsi-brain group were significantly higher compared to those in the Sham-IR/Brain group. The expressions of CXCL12, TGF-β1 and TNFα of the IR/Contra-brain group were significantly higher than those of the Sham-IR/Brain group. These results were expressed as mean ± SD and comparisons between groups were assessed by Student’s t-test.

Based on the results of the above trascriptome analysis (RNA-seq.), we performed validation qPCR for several genes. As shown in Fig. 5, significant increases were observed in the expressions of CXCL12, VEGF-A, TGF-β1 and TNFα in the IR/Ipsi-brain group at 3 months after radiation compared to the Sham-IR/Brain group. The expressions of CXCL12, TGF-β1 and TNFα in the IR/Contra-brain group were significantly higher than those of the Sham-IR/Brain group.

### Stimulation of genes and molecules in the tumor by irradiation

**Figure 6:**
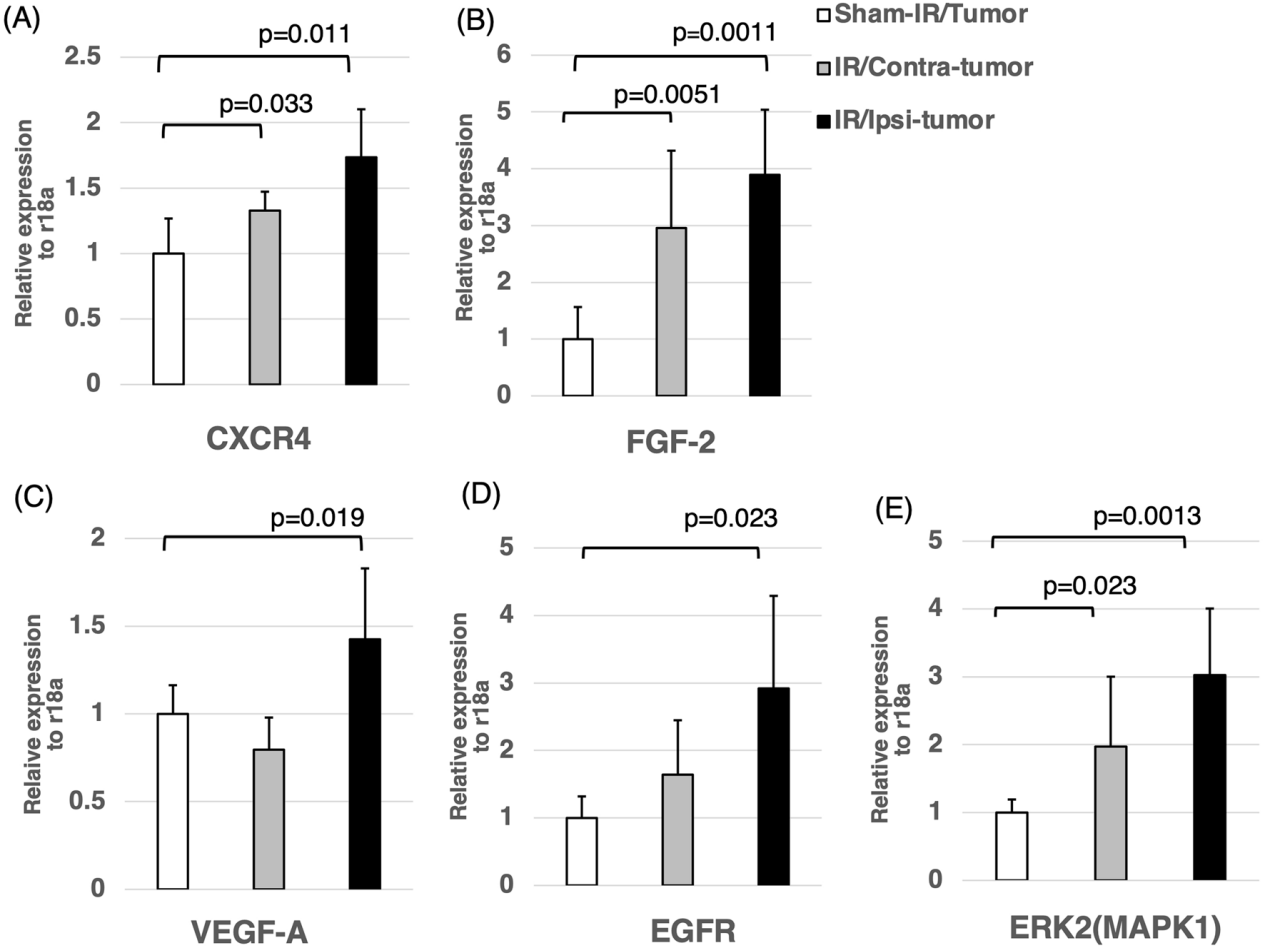
Expression of various cytokines in the IR/Ipsi-tumor, IR/Contra-tumor, and Sham-IR/Tumor groups. The expressions of CXCR4, FGF-2, VEGF-A, EGFR and ERK2 in the IR/Ipsi-tumor group were significantly higher than those in the Sham-IR/Tumor group. The expressions of CXCR4, FGF-2 and ERK2 in the IR/Contra-tumor group were significantly higher compared to those in the Sham-IR/Tumor group. These results were expressed as mean ± SD and comparisons between groups were assessed by Student’s t-test.

As shown in Fig. 6, significant increases were observed in the expressions of CXCR4, FGF-2, VEGF-A, EGFR and  ERK2 (MAPK1) in the IR/Ipsi-tumor group compared to the Sham-IR/Tumor group. The expressions of CXCR4, FGF-2 and ERK2 in the IR/Contra-tumor group were significantly higher than those in the Sham-IR/Tumor group.

## Discussion

In this study, we have demonstrated that the production of proinflammatory cytokine increased in the microenvironment of the parenchymal brain tissue (tumor bed) in the chronic phase after exposure to X-ray radiation. This effect is considered to be the microenvironment that is not suitable for the survival of tumor cells. However, the current study suggest that X-ray radiation made the microenvironment of brain tissue in the chronic phase suitable for the regrowth of glioma cell and invasion of the brain tissue.

First, compared with proteins extracted from rats that received sham exposure, proteins extracted from the radiation-exposed hemisphere promoted tumor growth in F98 glioma cells as well as tube formation in HUVECs. Interestingly, proteins extracted from non-exposed parts of the hemisphere in radiation-exposed rats showed similar effects, although they required high concentration of proteins. The results suggest an increase in the expression of proteins that promote the proliferative activity of tumor cells and neovascularity in brain tissue in the chronic phase after exposure to the radiation. In this experiment, local X-ray radiation to the hemisphere was confirmed through measuring the area of alopecia of the rat scalp (rats with areas of alopecia that crossed the midline were excluded from this study).

Then, we exposed the right hemisphere to local X-ray radiation and implanted F98 cells in the hemisphere at three months after exposure to the radiation. In this experiment, the implanted tumors did not receive X-ray radiation. Therefore, the difference in the behavior of the implanted tumors may be attributed to the differences in the microenvironment of the tumors. The overall survival (OS) of IR/Ipsi-tumor group after implantation of F98 glioma cells in the hemisphere previously exposed to X-ray radiation was significantly lower than that of the control (sham-IR/tumor) group (MST: 20.5 vs. 22.5, log-rank test p = 0.002). Moreover, interestingly, the OS of the group after implantation of F98 cells in the hemisphere that did not receive direct X-ray radiation (the IR/Contra-tumor group) was also significantly lower than that of the control group (MST: 20.5 vs. 22.5, log-rank test p = 0.003). The results of the analysis of proliferative activity, apoptosis rate (TUNEL staining), and density of tumor vessels (CD34 staining) in tumors harvested from the three groups showed a higher MIB1 index (by about 22%, p = 0.01) and blood vessel density (by about 40%, p = 0.045) and lower levels of TUNEL-positive apoptosis (by about 40%, p = 0.04) in tumors that were formed in the hemisphere after exposure to X-ray radiation (IR/Ipsi-tumor group) than in the control group. Conversely, compared to the control groups, tumors in IR/Contra-tumor group exhibited an increase in Ki-67 index by about 18% (p = 0.04) and a decrease in TUNEL-positive apoptosis by about 50% (p = 0.01), but no significant difference in the tissue blood vessel density. Among rodent GBM models, the F98 model is a disease model of tumor formation with extensive invasion of tumor-surrounding brain tissue^15^. The evaluation of the degree of invasion in the surrounding brain tissue showed a significant increase in the invasiveness in IR/Ipsi-tumor group than in sham-IR/tumor group. The above results suggest that X-ray radiation made the microenvironment of brain tissue in the chronic phase suitable for the regrowth of glioma cell and invasion of the brain tissue. It was reported that C6 glioma cells was irradiated before implantation to the rat brain, and the tumor showed activated infiltration into the brain^16^. Although the F98 glioma was not itself irradiated in this experiment, if the irradiated F98 had been transplanted into an irradiated animal, it is possible that the invasiveness might have been further increased. In addition, it will be interesting to study the survival outcomes of experiments in which irradiated and non-irradiated tumors are transplanted into the irradiated hemisphere. Therefore, to analyze the changes in the microenvironment of the brain tissue in the chronic phase after exposure to X-ray radiation at the molecular level, we performed transcriptomic analysis (RNA sequencing) of RNA extracted from the rat cerebral hemispheres (IR/Ipsi-brain and IR/Contra-brain groups) and the brain tissue harvested from sham group. In the analysis, we identified 52 genes exhibiting significantly high expression in the radiation-exposed hemisphere of rats previously exposed to radiation (IR/Ipsi-brain group) and in the non-exposed hemisphere of rats previously exposed to radiation (IR/Contra-brain group) compared to those in the control group (rats that did not receive radiation). Additionally, the pathway analysis of differentially expressed genes revealed significant changes in the pathways involved in tissue inflammation, such as cell adhesion molecules (CAMs), chemokine signaling pathway, leukocyte transendothelial migration, and cytokine-cytokine receptor interaction.

Further analysis of CXCL12, VEGF-A, TGF-β1 and TNFα using RT-PCR revealed a significant increase in the expression of the above-mentioned four genes except for VEGF-A in IR/Ipsi-brain and IR/Contra-brain groups compared to the control group. Conversely, the expression of VEGF-A was significantly high only in IR/Ipsi-brain group compared to the other groups. In previous studies, an increase in the expression of CXCL12, VEGF-A, TGF-β1 and TNF-α in the rat brain after a single high-dose X-ray radiation has been reported^17,18^. CXCL12 is a factor known to function as a ligand of CXCR4. It is known that CXCL12 is a chemokine that increases its production in the tissue as part of the inflammatory responses, and that the CXCL12/CXCR4 axis promotes the proliferative activity and invasiveness of tumor cells, including malignant glioma and other various cancers, with the expression of CXCR4, a receptor of CXCL12^19–22^. VEGF-A is a key factor involved in tumor neovascularization. Levin et al. reported that bevacizumab, a VEGF antibody, prevents radiation necrosis and related cerebral edema through reducing vascular permeability^23,24^. Additionally, bevacizumab has been reported to improve progression free survival in patients with glioma^25^. Furthermore, the activation of CXCR 4 has been reported to induce intratumoral expression of VEGF-A in glioma cells that respond to CXCL12^26,27^. TGF-β1 is an important factor involved in tumor growth and metastasis^28^. In vitro and in vivo experiments revealed that TGF-β1 released from microglial cells increased the invasiveness of glioma cells^29,30^. Moreover, TGF-β1 is a known factor that is also involved in the neovascularization of malignant tumors^31,32^. TNFα, a proinflammatory cytokine that plays an important role in the microenvironment of tumors has been associated with tumor invasion and metastasis. A previous study showed that TNFα activated MAPK/ERK signals, leading to an increase in the tumor invasiveness in breast cancer^33^. Additionally, TNFα has been reported to increase the invasiveness of C6 glioma as well^34,35^. Moreover, TNFα has been reported to increase CXCR4 expression in glioma cells^36^. A significant increase in the expression of the above-mentioned proinflammatory cytokine in the parenchymal tissue harvested from the hemisphere at three months after exposure to X-ray radiation in this study was consistent with previous studies in humans and animals^37–39^. An increase in the production of proinflammatory cytokine has been suggested to be due to the changes in the microenvironment of the parenchymal brain tissue (tumor bed) in the chronic phase after exposure to X-ray radiation.

Next, to analyze the effect of the microenvironment of the brain tissue (tumor bed) in the chronic phase after exposure to X-ray radiation on the implanted tumor cells at the molecular level, we performed sequencing of RNA extracted from glioma implanted in IR/Ipsi-tumor and IR/Contra-tumor groups as well as sham group. RT-PCR analysis of CXCR4, FGF-2, VEGF-A, EGFR, and ERK2 (MAPK1) revealed significantly higher expression of three genes (i.e., CXCR4, FGF-2, and ERK2) in IR/Ipsi-tumor and IR/Contra-tumor groups compared to the control groups. Conversely, the expression of VEGF-A and EGFR was significantly higher only in IR/Ipsi-tumor group compared to the other groups. As described above, an increase in the expression of CXCL12 in the brain after exposure to X-ray radiation may affect the implanted tumor cells, leading to an increase in the expression of CXCR4 in tumor cells. Additionally, glioma cells also secrete CXCL12, probably leading to the proliferation and neovascularization of glioma through the autocrine/paracrine mechanism involved in the CXCL12/ CXCR4 receptor ligand system. Analysis of the brain tumor model with increased tissue expression of CXCL12 after exposure to the radiation of glioma cells implanted in the brain compared with tumors implanted in the brain that did not receive radiation showed higher MIB1 expression, as a marker of proliferative activity (Fig. 3A), and higher infiltration in the periphery of tumor tissue (Fig. 3D). CXCR4 expression was also detected in vascular endothelial cells, and an increase in CXCL12 expression in the local tissue is known to promote neovascularization^40,41^. Additionally, extracellular CXCL12 promotes tumor angiogenesis through the production of VEGF-A by tumor cells^27^. Analysis of the number of vessels per unit area in tumor tissue also showed high blood vessel density of tumor cells implanted in the brain after exposure to the radiation (Fig. 3C). The expression levels of EGFR, a receptor that exhibits high expression in glioma tissues, have been significantly correlated with the malignancy of glioma^42^. It has been reported that CXCL12 activates EGFR ligands that are located downstream of CXCL12/CXCR4^43,44^. Our current study also showed a significant increase in EGFR expression in tumors implanted in the brain with high expression of CXCL12 in the chronic phase after exposure to the radiation (Fig. 6). Analysis of glioma cells implanted in rat brain after exposure to the radiation compared to those in the control groups (i.e., rats that did not receive X-ray radiation) revealed a significant increase in the production of FGF-2 by the tumor cells and a significant decrease in the rate of differentiation of apoptotic cells in the tumor tissue. It is known that an increase in FGF-2 expression induced through autocrine production leads to increased proliferative activity, reduced apoptosis, and increased neovascularization, resulting in the maintenance of glioma stem cells^45,46^. Furthermore, the activation of these growth factors and MAPK (MEK-ERK) pathway, an intercellular signaling pathway involved in cell growth located downstream of their receptors, may lead to an increase in the production of glioma cells^47^. High expression of ERK has been confirmed in glioma cells implanted in rat brain after exposure to the radiation.

The results of this study suggest that the microenvironment (tumor bed) round the parenchymal brain tissue, especially humoral factors (mainly cytokines), at several months after exposure to X-ray radiation, becomes suitable for the replication of tumor cells that survived after X-ray treatment, mainly through modifying the phenotype of the tumors (e.g., proliferative activity of glioma, antiapoptotic effects, invasiveness, and tumor angiogenesis). In this study, glioma cells implanted in the brain did not receive radiation. Therefore, the changes in tumor cells (e.g., proliferative activity, apoptosis, and neovascularization) observed in this study may be attributed to the differences in the microenvironment (tumor bed) of parenchymal brain tissue where glioma cells were implanted. This experiment was able to demonstrate only the effect of radiation-induced late brain injury on tumors. More interestingly, the results of this study revealed a significant increase in the proliferative and antiapoptotic effects on tumors that were also formed in the non-exposed hemisphere of rats previously exposed to X-ray radiation. Such changes in the tissue microenvironment (tumor bed) in the chronic phase after exposure to the radiation may also be observed outside the radiotherapy field. Sequencing of RNA extracted from the brain tissue at three months after exposure to X-ray radiation also ranked high in “the chemokine signaling pathways” as the pathways of differentially expressed genes (Table.1). Therefore, the above-mentioned effects on area outside the radiotherapy field may be due to humoral factors, such as secretory growth factors and cytokines, that are activated through chronic tissue inflammation induced upon exposure to X-ray radiation. In fact, the validation of interstitial expression of CXCL12, TGF-β1, TNFα and others, using quantitative PCR showed a significant increase in the expression of these humoral factors in the non-exposed hemisphere of rats previously exposed to X-ray radiation (Fig. 5). Furthermore, the results suggest that the tissue proteins extracted from the non-exposed hemisphere of rats previously exposed to X-ray radiation increased the proliferative activity of glioma cells in vitro and promoted the tube formation in vascular endothelial cells (Fig. 1C).

Currently, among chemotherapeutic agents used for the prevention of the recurrence of tumor after radiotherapy, only alkylating agents (e.g., TMZ, ACNU/BCNU) exhibited evidence of improved prognosis in patients with glioma^3,48,49^. Cytokines related to angiogenesis and inflammation are secreted during the tissue repair process in resection margins after glioma surgery, which suggest that they may be involved in the recurrence of residual tumors^50^. In this experiment, the same response was observed in the irradiated brain, which may have contributed to the growth and angiogenesis of the implanted tumor. Therefore, to develop new therapies to inhibit glioma recurrence, it is not enough to use drugs that directly damage tumor cells, but also it is important to control cytokines in the tumor microenvironment. Recently, tumor immunotherapy using PD-1 inhibitors has also been focused. Zeng et al. suggested that radiosurgery plus PD-1 blockade generates robust antitumor activity against primary intracranial gliomas^51^. They showed that the combination of SRS and PD-1 enhanced the anti-tumor immune response while minimizing the immunosuppressive response to irradiation of the normal brain. The pathway analysis in this study (Table 1) showed the activation of chronic inflammation and autoimmune response in brain tissue after X-ray irradiation. In this experiment, it is unclear to the extent to which anti-tumor immunity effect acted on the transplanted glioma cells, but it is considered to have acted in the direction of promoting tumor growth in the irradiated brain tissue as a whole.

This study has several limitations. First, in this study, we did not assess the volume of implanted tumors using MRI or contrast-enhanced CT. It is because the assessment of the volume of implanted tumors using imaging requires anesthesia for at least 30 min that may have affected the prognosis of rats. Second, in this study, the evaluation was conducted only at three months after the exposure to X-ray radiation. If the tumors were implanted at later stage after exposure to X-ray radiation, the proliferative activity and malignancy of glioma must have been higher than those documented in this study due to radiation-induced changes in the secretion of cytokines.

Third, it is to be noted that the microenvironment of the irradiated rat brain is different from that of the recurrent tumor. In clinical practice, when radiotherapy for malignant gliomas is performed, the irradiated area includes the tumor and the surrounding normal brain. Therefore, we should consider the effects of cytokines secreted by the recurrent tumor in the microenvironment. The generation of a glioma model with stable OS required the implantation of about 10,000 tumor cells. Nevertheless, it was difficult to generate a brain tumor model with glioma recurrence within several months after exposure to the radiation because the OS of the brain tumor model rats was less than four weeks. Therefore, we transplanted glioma cells to the brain after X-ray irradiation in this study. Lastly, a single radiation dose of 65 Gy is relatively higher than that used for radiotherapy of patients with malignant glioma. Postoperative radiotherapy (stupp regimen) for malignant gliomas is generally performed in 60 Gy/30 fractions. Since this study was a single dose irradiation, it is expected that the brain damage and inflammatory response associated with irradiation is stronger than that of fractionated irradiation. Single fraction might have led to increased production of pro-inflammatory cytokines in brain tissue after X-ray irradiation. Additionally, due to the difference in ischemic tolerance between rats and humans, the effect of radiation on rat cortex is lower than that on human cortex. Studies on radiation-induced late brain injury have reported that a simulation of changes in the chronic stage in a rodent model requires exposure to high-dose X-ray radiation of about 50–60 Gy^52–54^. Although data are not shown in this paper, in our pilot study, we generated a rodent model of radiation-induced late brain injury that exhibited recurrence within 6 months induced through X-ray radiation of 65 Gy. The model exhibited an increase in the expression of chemokines and immunological response-related activation of microglial cells. Therefore, tumors were implanted at three months after a single exposure to the radiation of 65 Gy.

Unlike previous findings on the tumor bed effect (claiming that tumor bed exhibits tumor inhibitory effect), the current study suggests that the microenvironment around the brain tissue in the chronic phase after exposure to X-ray radiation becomes suitable for glioma cell growth and invasion. In the future studies, development of new therapeutic agents to delay the recurrence of glioma should be conducted focusing on the effect of radiation injury on brain tissue after exposure to the radiation (i.e., tumor bed).

## Methods

### Cell line

The rat F98 glioma cell line was obtained from ATCC (Manassas, VA) and grown in Dulbecco’s Modified Eagle Medium (DMEM) supplemented with 10% fetal bovine serum and penicillin at 37 °C in an atmosphere of 5% CO2. These materials for the culture medium were purchased from Gibco Invitrogen (Grand Island, NY, USA).

### Animal experiment A: X-ray irradiation to the rat brain

All of the experimental procedures used in this study were approved by the Animal Review Board and Ethical Committee of Osaka Medical College (Approval No.: 27058). This study was conducted in accordance with the 'Ethics Declaration' for the ARRIVE guidelines' (https://arriveguidelines.org). Eight-week-old, male Fischer 344 rats weighting between 270–320 g (Japan SLC, Inc. Shizuoka, Japan) were anesthetized with an intraperitoneal injection of a mixture of medetomidine 0.15 mg/kg, midazolam 2 mg/kg, and butorphanol 2.5 mg/kg.

**Figure 7:**
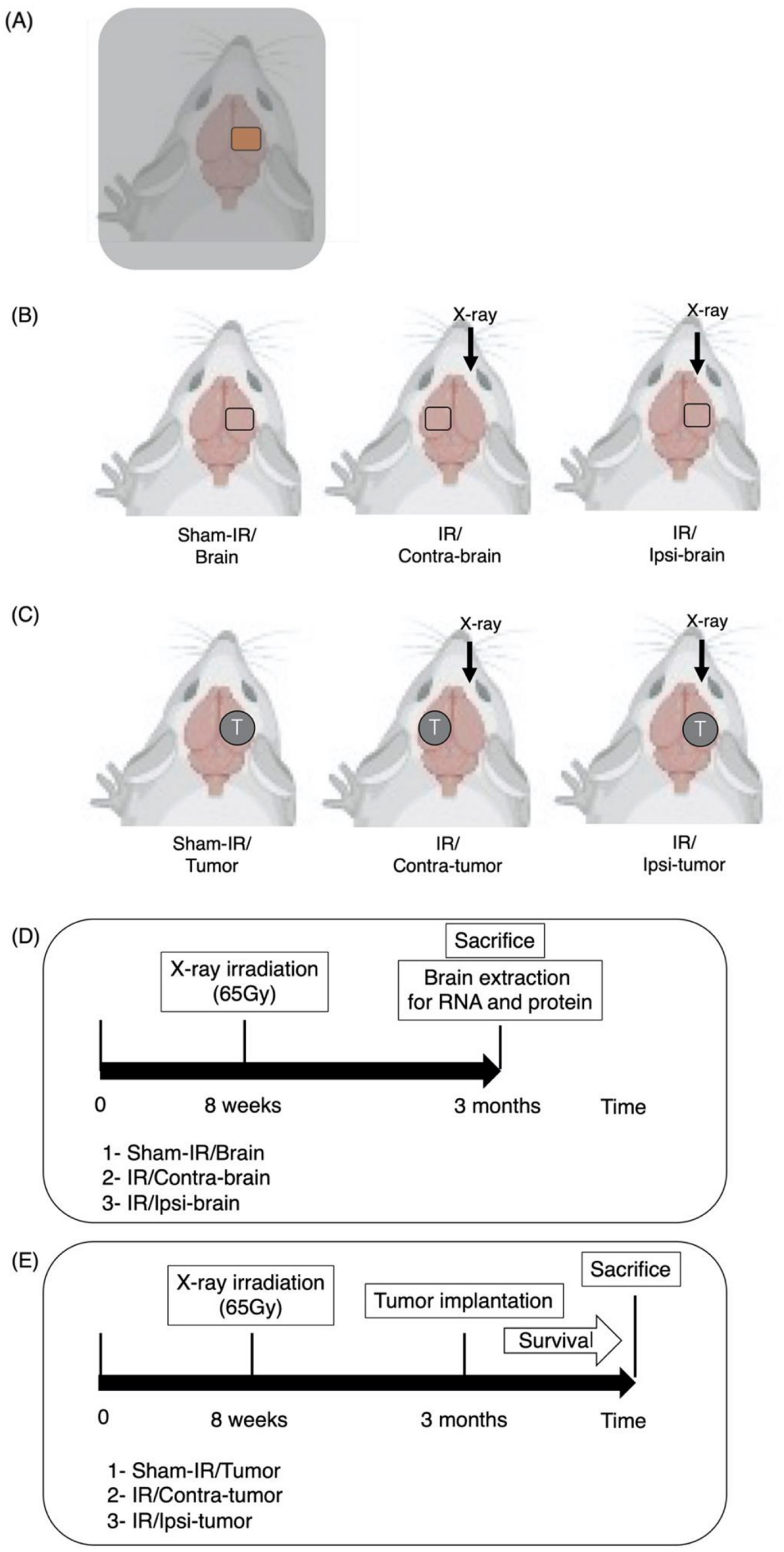
(**A**) Schema of X-ray irradiation to the rat brain (created with BioRender.com). The whole body of rat was covered with a lead plate with a 1 cm square window, and only a part of the right cerebral hemisphere was irradiated with X-rays. (**B**) Treatment schema of the three groups of male Fisher rats for in vitro study (created with BioRender.com). X-ray: X-ray irradiation. IR/Ipsi-brain group: X-ray was irradiated only a part of the right cerebral hemisphere and the tissue of the right hemisphere was used. IR/Contra-brain group: X-ray was irradiated only a part of the right cerebral hemisphere and the tissue of the left hemisphere was used. Sham-IR/Brain group: no irradiation was performed and the tissue of the right hemisphere was used. (**C**) Treatment schema of the three groups of Fisher rats implanted tumor for in vivo study (created with BioRender.com). T: implanted tumor. IR/Ipsi-tumor group: F98 cells were transplanted to the right hemisphere after irradiation to the right hemisphere. IR/Contra-tumor group: F98 cells were transplanted to the left hemisphere after irradiation to the right hemisphere. Sham-IR/Tumor group: F98 cells were transplanted to the right hemisphere without irradiation. (**D**) Timeline describing the timing of irradiation, sacrifice and brain extraction in vitro assay. Three months after the X-ray irradiation, we extracted the tissue protein and RNA from rat brain. (**E**) Timeline describing the timing of irradiation, tumor implantation and sacrifice for in vivo assay. Three months after the irradiation, F98 glioma cells were implanted into the cerebral hemisphere of rats.

During X-ray irradiation, the 5 mm-thick lead shield with a 1 cm square window were used to protect the whole body of the rats (Fig. 7A). For rats in the irradiation groups (IR: n = 8), 100-kV X-ray was irradiated to the right cerebral hemisphere through the 1 cm square window, with a single dose of 65 Gy using a linear X-rat accelerator (SOFTEX M-150WE; Kanagawa, Japan). The irradiation area was determined with the bregma as reference points. The sham-irradiation was performed for rats in the control group (Sham-IR: n = 7).

### RNA and protein extraction from the irradiated rat brain

Three months after the X-ray irradiation, the rats were euthanized to obtain their brain tissue. After the brains were divided along the midline, the tissue protein and whole RNA were separately extracted from the right (IR/Ipsi-brain: n = 8) and left cerebral hemispheres (IR/Contra-brain: n = 8) of the animals irradiated on the right brain as mentioned in Fig. 7B,D. As a control, we prepared proteins and total RNA extracted from the right cerebral hemisphere of the sham-irradiated animals (Sham-IR/Brain: n = 7). The RNA was extracted using miRNeasy Mini Kit (Qiagen, Hilden, Germany), and cDNA library preparation was performed using a SuperScript VLIO cDNA Synthesis kit (Thermo Fisher Scientific, Cleveland, USA) according to the manufacturer’s instructions. The brain tissue protein was extracted with the 3-min total protein extraction kit for animal tissues (101 Bio, Palo Alto, CA). The concentrations of the extracted protein were determined by the PierceTM BCA Protein Assay Kit (Thermo Scientific-Life Technologies, Waltham, MA).

### In vitro cell proliferation assay using F98 glioma cells

The proliferation ability of the tumor cells was analyzed by a WST-8 assay. F98 cells were seeded in 96-well plates at a density of 10,000 cells/well in 100 μl of culture serum-free DMEM. At 24 h after this pre-incubation, 100 μl of medium containing the extracted brain protein with 1.5, 3.0, 6.0 and 12 μg/ml was added to the wells. To study the effect of AMD3100, a CXCR4 antagonist, glioma cells were incubated with 10 μM AMD3100. 72 h after the incubation, 10 μl of WST-8 labeling reagent (Cell Counting Kit; Dojindo Laboratories, Kumamoto, Japan) were added to each well and the absorbance at 450 nm was measured with the corona grating microplate reader (Hitachi High-Tech Corporation, Japan).

### In vitro tube-formation assay using HUVEC

For the in vitro tube-formation assays, 96-well plates were coated with growth-reduced factor Matrigel (BD Biosciences, catalog number: 354230) on ice and incubated at 37 °C for 2 h until the Matrigel became solid. Human umbilical vein endothelial cells (HUVECs) (Life Technologies, Invitrogen, catalog number: C-003-5C) were seeded in 96-well plates at a density of 20,000 cells/well and cultured in 150 μl of medium containing 3.0 or 6.0 μg/ml of the cerebral proteins extracted the above. After a 3-h (incubation in 5% CO2 at 37 °C, images were taken with at a magnification of × 100 (10 ocular × 10 objective), and the lengths of the tubes in four random fields were quantified by Image J software version1.52. (U.S. National Institutes of health, Maryland, USA).

### Animal experiment B: glioma cell transplantation into the irradiated rats

#### Animal groups and study design

Thirty-two male Fischer 344 rats (8-week-old, weighting between 270 and 320 g) were used.

65-Gy of X-ray was irradiated to the right cerebral hemisphere of 20 rats as described above, and sham-irradiation was performed for 12 rats to prepare a control group.

Three months after the irradiation, the 20 rats were randomly divided into two groups (IR/Ipsi-tumor or IR/Contra-tumor) as mentioned in Fig. 7C. In “IR/Ipsi-tumor” group (n = 12), the F98 glioma cells were implanted to the right cerebral hemisphere of the irradiated animals. On the other hand, in the “IR/Contra-tumor” group (n = 8), F98 cells were implanted to the left hemisphere of the irradiated animals. All the sham-irradiated animals (n = 12) were implanted with F98 cells into right hemisphere (Sham-IR/Tumor group). The rats were observed daily for body weight and neurological deficits (n = 5). They were euthanized by inhalation of Sevoflurane according to standardized criteria, > 20% weight loss for 2 days, and the brains were removed for the following analyses (Fig. 7E).

### Tumor implantation

At the chronic phase (3 months) after the X-ray irradiation, F98 glioma cells were implanted into the cerebral hemisphere of rats in the above three groups. Under anesthesia, the rat’s head were fixed with a stereotactic frame (Model 900; David Kopf Instruments, Tujunga, CA, USA), and a small burr hole was made on the skull with an electric drill at 1 mm anterior and 3 mm lateral of the bregma. 1 × 10^4^ of F98 cells were suspended in 10 μl of DMEM and injected at a rate of 1 μl/min into the brain at a depth of 5 mm from the skull burr hole, using a 25-μl Hamilton microsyringe with a 26-gauge needle (model 1700 RN, Hamilton Bonaduz AG, Bonaduz, Switzerland) and a micro-injector (WPI model UMP3, Sarasota, FL, USA). After the injection was complete, the burr hole was sealed with bone wax.

### Immunohistochemical analyses for the tumor

#### Cell proliferation index

The primary antibody, Ki-67 (clone MIB-1; dilution of 1:100) (Abcam, Cambridge, MA, USA), and a universal secondary antibody (dilution of 1:300) (Roche, Basel, Swizerland) were used. The cell proliferation index was calculated by taking five random pictures of each slide at a magnification of 200 × (10 ocular × 20 objective). The index was calculated as the number of Ki-67-stained cells divided by the total number of cells. HematoxylinIIwas used for the counterstaining for cell nuclei.

#### Apoptotic index

To detect the cell apoptosis, the terminal deoxynucleotidyl transferase (TdT)-mediated dUTP nick end labeling (TUNEL) assay was performed using an ApopTag Peroxidase In Situ Apoptosis Detection Kit (Cosmo Bio, Tokyo, Japan). The apoptotic index was evaluated by counting the number of TUNEL-positive cells over the total number of cells at a magnification of 200 × (10 ocular × 20 objective) in five random fields.

#### Microvascular density (MVD) index

The primary antibody, CD34 (dilution of 1:2500) (Santa Cruz Biotechnology, Santa Cruz, CA, USA), was used with a universal secondary antibody (dilution of 1:300) (Roche, Basel, Swizerland). The MVD index was calculated by taking five random pictures of each slide at a magnification of 200 × (10 ocular × 20 objective). The index was calculated as the number of CD34-stained cells divided by the total number of cells.

### Evaluation of glioma invasiveness

F98 rat glioma model shows tumor cell invasion into normal brain parenchyma at the tumor periphery. Implanted brain tumors were removed from the three groups; IR/Ipsi-tumor, IR/Contra-tumor, and Sham-IR/Tumor groups, fixed with formalin and embedded in paraffin. The FFPE blocks were sliced and stained with hematoxylin and eosin. The invasion index was evaluated by taking pictures of each slide at a magnification of 50 × (10 ocular × 5 objective). The index was calculated as the total area of glioma crossing tumor rim divided by the field of tumor regions in serial sections using *Image J* software^55^. The invasive tumor ratio of the Sham-IR/Tumor group was defined as 1.0.

### RNA extraction

Total RNA was extracted and prepared cDNA from the F-98 cell implanted tumors in the three groups; IR/Ipsi-tumor, IR/Contra-tumor, and Sham-IR/Tumor as described above.

### RNA-sequencing

Whole transcriptome sequencing was applied to the RNA samples of brain tissues in triplicate assay (n = 3 for each group), with use of on an Illumina HiSeq 2500 platform in a 75-base single-end mode. Illumina Casava ver.1.8.2 software was used for base calling. Sequenced reads were mapped to the rat reference genome sequences (rn6) using TopHat ver. 2.0.13 in combination with Bowtie2 ver. 2.2.3 and SAMtools ver. 0.1.19. The number of fragments per kilobase of exon per million mapped fragments (FPKMs) was calculated using Cuffnorm ver. 2.2.1. The raw data have been deposited in the Gene Expression Omnibus database of the U.S. National Center for Biotechnology Information (NCBI).

As compared to Sham-IR/Brain, the differentially expressed genes (DEGs) in IR/Ipsi-brain and IR/Contra-brain were obtained using a threshold of 0.05 for statistical significance (p-value) and a log fold change of expression with absolute value of at least 1.5. To identify significantly altered pathways, we used an analysis software, “*iPathwayGuide*” (Advita Corporation, Plymouth, MI, USA; info@advaitabio.com). These DEGs were analyzed with the impact analysis method^56–58^, in the context of pathways obtained from the Kyoto Encyclopedia of Genes and Genomes (KEGG) database (Release 81.0 + /01–20, Jan 17)^59,60^, and gene ontologies from the Gene Ontology Consortium database (2016-Sep26)^61,62^. (See Supporting information: Methods for pathway analysis).

### Real-time quantitative PCR (qPCR)

Total RNAs were reverse-transcribed into cDNA with a SuperScript VLIO cDNA Synthesis kit (Thermo Fisher Scientific, Cleveland, USA). The validation gene for the quantitative polymerase chain reaction (qPCR) was selected based on the results of RNA-sequencing. A quantitative real-time PCR was performed with TaqMan probes and primers using the LightCyclerII (Roche Applied Science, Penzberg, Germany). The PCR conditions were as follows: a single denaturation cycle at 95 °C for 10 min, followed by 45 amplification cycles of 95 °C for 10 s and 60 °C for 25 s. The relative expression ratio was calculated after normalization with reference to the expression of the housekeeping gene 18S RNA. The PCR primers were designed by the Roche Universal Probe Library. The expression of cytokines such as C-X-C motif chemokine 12 (CXCL12), vascular endothelial growth factor-A (VEGF-A), tumor growth factor β1 (TGF-β1), tumor necrosis factor alpha (TNFα), C-X-C chemokine receptor type 4 (CXCR4), fibroblast growth factor-2 (FGF-2), epidermal growth factor receptor (EGFR) and extracellular signal-regulated kinase2 (ERK2) was assessed by qPCR.

### Statistical analysis

Data are expressed as the mean ± standard deviation. Statistical analysis of the groups' MST values was performed using the Wilcoxon log-rank test, and other results were analyzed using Student's t-test. Values of p < 0.05 were considered statistically significant.

### Ethics approval and informed consent

All applicable international, national, and institutional guidelines for the care and use of animals were followed. Informed consent was obtained from all individual participants included in the study.

## Supplementary Information

Supplementary Information.

## Data Availability

All data generated during the current study are included in this article and are available from the corresponding author on reasonable request.

## Abbreviations

COX-2: Cyclooxygenase-2
CXCR-4: C-X-C chemokine receptor type 4
CXCL-12: C-X-C motif chemokine 12
DMEM: Dulbecco’s Modified Eagle Medium
EGFR: Epidermal growth factor receptor
ERK2: Extracellular signal-regulated kinase2
FGF-2: Basic fibroblast growth factor-2
GBM: Glioblastoma multiforme
IL-1β: Interleukin-1β
MMP-2: Matrix metalloproteinase-2
MST: Median survival time
MVD: Micro vascular density
qPCR: Quantitative polymerase chain reaction
TGF-β1: Tumor growth factor β1
TNFα: Tumor necrosis factor alpha
TUNEL: Terminal deoxynucleotidyl transferase (TdT) -mediated dUTP nick end labeling
VEGF-A: Vascular endothelial growth factor-A
