# Supplementary material for: Chronic pathophysiological changes in the normal brain parenchyma caused by radiotherapy accelerate glioma progression
Source: Sci Rep. 2021 Nov 11;11:22110. doi: 10.1038/s41598-021-01475-0 (PMC8585920; doi:10.1038/s41598-021-01475-0)
Supplement: Supplementary file 1 — Supplementary Information. [file 41598_2021_1475_MOESM1_ESM.docx]

**Supplementary Table1:** The list of using primer for qPCR

| **Gene Name** | **Sequences** |
| --- | --- |
| CXCR4 | 5’-GCTGGAGAGCGAGCATTG-3’  5’-CCTGTTGAAGTTTTCGTTTTCA-3’ |
| CXCL12 | 5’-CCCTGCCGATTCTTTGAG-3’  5’-GCTTTTCACCTTGCAACA-3’ |
| EGFR | 5’-TGCACCATCGACGTCTACAT-3’  5’-AACTTTGGGCGGCTATCAG-3’ |
| ERK2 | 5’-GGGTTCTTGACAGAGTATGTAGCC-3’  5’-ACCAAATATCAATGGACTTGGTATAA-3’ |
| FGF-2 | 5’-TCTTCCTGCGCATCCATC-3’  5’-GCTTGGAGCTGTAGTTTGACG-3’ |
| S18a | 5’-CCAGAAGGATGCTCACCAA  5’-GAGCCCAGCGAGTCAGATAG |
| TGF-β1 | 5’-TGGAGCAACATGTGGAACTC  5’-GTCAGCAGCCGGTTACCA |
| TNFα | 5’-TGAACTTCGGGGTGATCG  5’-GGGCTTGTCACTCGAGTTTT |
| VEGF-A | 5’-GAGTTAAACGAACGTACTTGCAGA  5’-TCTAGTTCCCGAAACCCTGA |

**Supplementary Figure 1:** Significantly impacted pathways in IR/Ipsi-brain and IR/Contra-brain.

The Venn diagram indicates the number of the significantly impacted pathways in IR/Ipsi-brain and IR/Contra-brain groups, as compared to the control group (Sham-IR/Brain).

**Supplementary information**

**Methods of the pathway analysis used in “*iPathwayGuide*”** (Advita Corporation, Plymouth, MI, USA)

iPathwayGuide scores pathways using the Impact Analysis method (Draghici et al., 2007; Tarca et al., 2009, Khatri et al., 2007). Impact analysis uses two types of evidence: i) the over-representation of differentially expressed (DE) genes in a given pathway and ii) the perturbation of that pathway computed by propagating the measured expression changes across the pathway topology. These aspects are captured by two independent probability values, pORA and pAcc, that are then combined in a unique pathway-specific p-value. The underlying pathway topologies, comprised of genes and their directional interactions, are obtained from the KEGG database (Kanehisa et al., 2000; Kanehisa et al., 2014).

The first probability, pORA, expresses the probability of observing the number of DE genes in a given pathway that is greater than or equal to the one observed by random chance (Draghici et al., 2003; Draghici 2011). Let us consider there are N genes measured in the experiment, with M of these on the given pathway. Based on a priori selection of DE genes, K out of M genes were found to be differentially expressed. The probability of observing exactly x differentially expressed genes on the given pathway is computed based on the hypergeometric distribution:


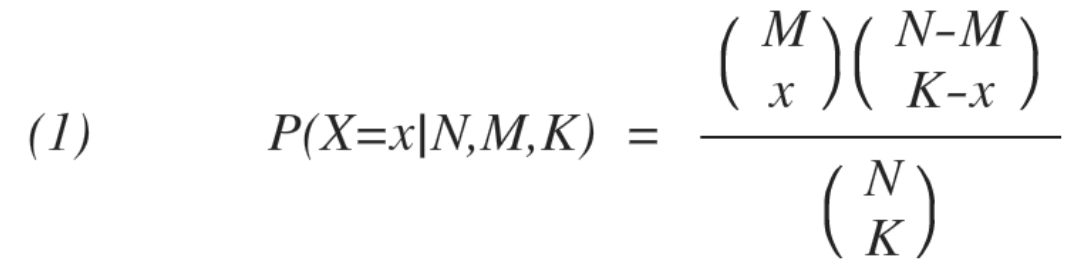


Because the hypergeometric distribution is discrete, the probability of observing fewer than x genes on the given pathway just by chance can be calculated by summing the probabilities of randomly observing 1, 2, ..., up to x-1 DE genes on the pathway:


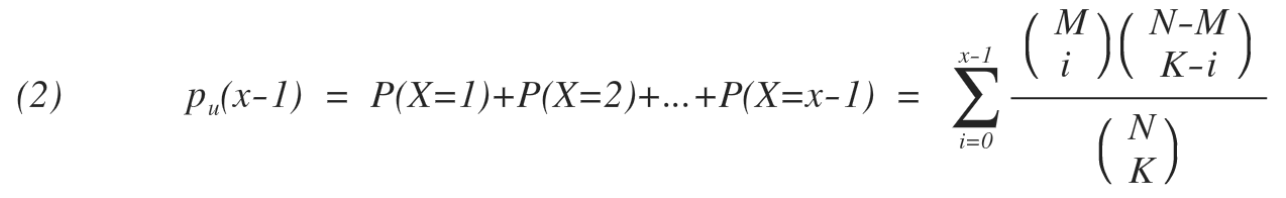


iPathwayGuide calculates the probability of randomly observing a number of DE genes on the given pathway that is greater than or equal to the number of DE genes obtained from data, by computing the over-representation p-value: pORA = po(x) = 1 - pu(x-1):


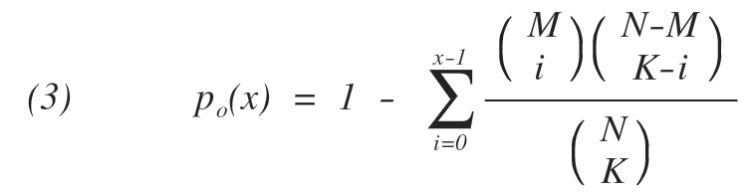


The second probability, pAcc, is calculated based on the amount of total accumulation measured in each pathway. A perturbation factor is computed for each gene on the pathway using:


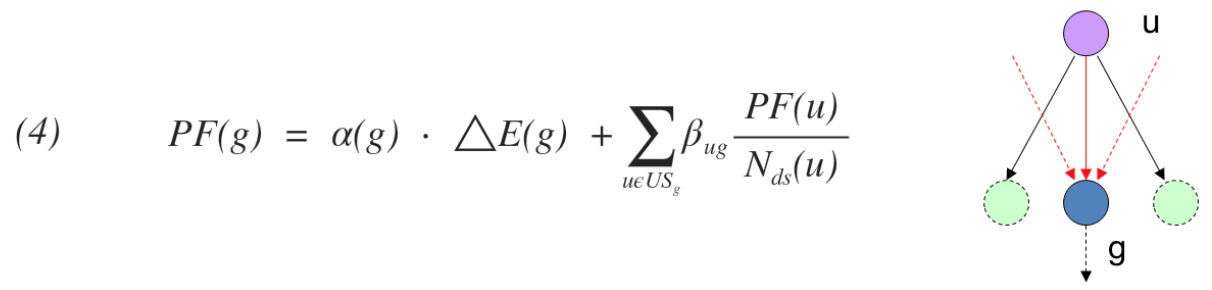


In Equation 4, the term ΔE(g) represents the signed normalized measured expression change of gene g, and α(g) is a priori weight based on the type of the gene. The last term is the sum of the perturbation factors of all genes u, directly upstream of the target gene g, normalized by the number of downstream genes of each such gene Nds(u). The value of βug quantifies the strength of the interaction between genes g and u. The sign of β represents the type of interaction: positive for activation-like signals, and negative for inhibition-like signals. Subsequently, iPathwayGuide calculates the accumulation at the level of each gene, Acc(g), as the difference between the perturbation factor PF(g) and the observed log fold-change:


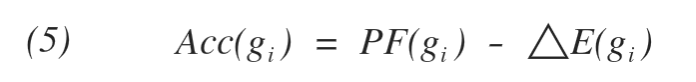


All perturbation accumulations are computed at the same time by solving the system of linear equations resulting from combining Equation 4 for all genes on a given pathway. Once all gene perturbation accumulations are computed, iPathwayGuide computes the total accumulation of the pathway as the sum of all absolute accumulations of the genes in a given pathway. The significance of obtaining a larger total accumulation (pAcc) just by chance is assessed through bootstrap analysis.

The two types of evidence, pORA and pAcc, are combined into one final pathway score by calculating a p-value using Fisher's method. This p-value is then corrected for multiple comparisons using false dicovery rate (FDR) or Bonferroni corrections. Bonferroni is simpler and more conservative of the two (Bonferroni, 1935; Bonferroni, 1936). It reduces the false discovery rate by imposing a stringent threshold on each comparison adjusted for the total number of comparisons. The FDR correction has more power but only controls the family-wise false positives rate (Benjamini and Hochberg, 1995; Benjamini and Yekutieli, 2001).
